# Criminal Responsibility of the Insane

**Published:** 1906-08

**Authors:** F. E. Daniel

**Affiliations:** Austin, Texas., Vice-Chairman, Section Psychology, Medico-Legal Society. New York, Etc.


### TEXAS MEDICAL JOURNAL.

Established July, 1885.

F. E. DANIEL, M. D., - - - - Editor, Publisher and Proprietor.

PUBLISHED MONTHLY.—SUBSCRIPTION $1.00 A YEAR.

VOL. XXII. AUSTIN, AUGUST, 1906. No. 2.

The publisher is not responsible for the views of contributors.

### Criminal Responsibility of the Insane:

WITH ESPECIAL REFERENCE TO THE CASE OF EUGENE BURT.*

BY F. E. DANIEL, M. D., AUSTIN, TEXAS.,

Vice-Chairman, Section Psychology, Medico-Legal Society, New York, Etc.

There is a widespread and growing conviction that the reform of the criminal law is a pressing necessity. The inefficiency of our penal statutes is due to the fact that they are enacted, for the most part, by men who make no pretension to scientific knowledge, and are notoriously averse to being advised. The unwise policy that obtains in some States of paying legislators day-laborers' wages is largely responsible for this. Such pay is not calculated to command a high order of lawmaking talent. The legislature is composed of representative citizens,—farmers, merchants, mechanics, lawyers,—most of whom have no knowledge of science, and do not want to be told; they can not be prevailed upon to make any reform in accord with the teachings of science. It is this element that defeats all efforts of the medical profession in Texas to secure legislation in the interest of public health. That the jurisprudence of insanity is far behind the present status of medical science on this subject is very generally admitted; it belongs to a past age, and is therefore not adapted to the needs of a later-day civilization. On this head Judge Abbott† says:

"The rude division into 'idiots' and 'lunatics' of two centuries ago survives in jurisprudence to-day. * * * Jurisprudence has had no peculiar method of studying the subject, but has been ac-

*This paper is substantially a lecture which was delivered by invitation before the Law School of the University of Texas in May, 1897. Reproduced by request. From *The Arena*, August, 1898.

†"Reference Handbook of Medical Sciences."

customed to follow the course of medical science, and to accept, sometimes indeed only after long hesitation and inquiry, the results which skillful and experienced alienists have united in declaring established."

This is a remarkable statement. What other course than that of medical science should our lawmakers follow in legislating upon a subject better understood by physicians than by any other class of investigators? From what other source is it to be expected that jurisprudence would derive the information necessary to guide them in settling questions involving the sanity of a supposed offender?

"The English law," says Mr. Tracy Becker,* "recognizes two states of mental disease: 1st, 'Dementia Naturalis,' and 2nd, 'Dementia Adventitia,' under which head general insantiy is included."

There are forty-four forms of insanity known to alienists.

My convictions on this subject were greatly strengthened by witnessing in Austin the trial of a man for the murder of his family under circumstances of peculiar atrocity; a man evidently insane. That case forms the basis of this criticism. The facts as elicited at the trial detailed below, were admitted by the defense notwithstanding that there were no witnesses, and that all the evidence as to the act itself was circumstantial. I quote from brief of defendant:

"On the night of July 24, 1896, defendant (W. E. Burt, white, native Texan, age 27) and his wife were at home at half-past eight or nine o'clock, when by the nurse the younger child was delivered to him, and the elder to the wife. After the lapse of a little while he went to the dining-room, filled a bottle with milk for such younger child, went upstairs to the room where he and his wife and children slept, leaving the wife, the elder child, and the servant in the lower rooms; this was the last of the younger child ever seen alive. After a time the servant departed, and did not return until 11 o'clock. All was quiet in the house at that time. A day or two before this he was seen coming from the stable with a grass sack in his hand which contained something. At some hour of the night, in their bedroom, the defendant killed his wife and two children by striking each of them in the right temple and side of the face with a hatchet, crushing the bones of the face, and fracturing the skulls. He then tightly tied around the throat of each a handkerchief, sufficiently so to produce stran-

*Withaus and Becker's "System of Medical Jurisprudence.'

gulation and suffocation; then enveloped the body of the wife, except the feet, in a blanket, and wound around the blanket ropes so as to keep the blanket in place and the body enveloped. He tied the hands and feet of the two children with wires and other ligatures, they being in their nightclothes. He then conveyed the bodies, by some means, in his arms (or by lowering them from a window, or through it, casting them out), from the upstairs room to the lower floor, to an underground cistern in the basement of the house, and cast the three bodies therein, and then nailed down the top of the cistern, which had been ripped off to admit the bodies. There was water in the cistern sufficient to submerge the bodies; the water in the cistern was in daily use by the household thereof. He took the handle of the cistern pump and secreted it. [Not a sign or stain of blood was seen anywhere.]

"The servant returned about 11 o'clock and slept in the house, but heard no noise except a faint dreamlike remembrance of hearing a child cry. The next morning at about 7 o'clock he rapped at the servant's door, awakening her, and requesting her to go to market, a thing she was not in the habit of doing. He was not seen again until the servant returned from market. On her return she took the teakettle, purposing to fill it with water, and in taking hold of it made a noise, when defendant said to her, 'Don't use the water from the cistern, as a cat fell in there last night.' Some questions about the wife and children arose, when he said that he had had some trouble in the night, and had sent them to San Antonio on the 5 o'clock train, but that they would be back Tuesday or Wednesday and everything would be in readiness to go keeping house at the Scott place. His breakfast being prepared, defendant gave a note to the servant to be carried to a cartman, directing him to go to the store of defendant's brothers and procure and bring to the house some boxes; he also gave her some money to buy some nails and bring to him; all of which was done as directed; and the cartman, on bringing the boxes, was requested to return at 3 or 4 o'clock. He ate his breakfast; he sent the servant with a note to a second-hand furniture man to come and look at the furniture and other household effects. He came and looked at the effects, and asked the price wanted, and was told one hundred and fifty dollars, but finally he agreed to take sixty-five dollars, and the trade was consummated at those figures, and the goods delivered.

"During the day the bloody clothing, sheets, bolsters, and pillows, and other blankets, comforts, all more or less bloody, a bloody hatchet, the hats and bonnets of the wife and miscellaneous clothing of the children (not bloody), bloody cotton from the mattress, portions of the ticking from a mattress, all bloody, were all packed in the packing boxes and nailed up, and at 4 o'clock delivered to the cartman to be conveyed to the transportation office for shipment from a fictitious person to a fictitious person in Houston, Texas. The addresses on the boxes were written in a feigned handwriting by the defendant. During the day he had various money transactions with different persons, wrote various notes, tore some up, and others were delivered to the persons to whom written. He was in or about the house the greater part of the day. In the evening the milkman came, whom defendant met at the door and said, 'This is the milkman,' got a pitcher for the milk, and told him the family had moved to 912 Rio Grande Street (there being no such street number), and that the next day he, the milkman, would find in the milk pitcher two tickets instead of one. At that time he appeared weary, as if having been hard at work, in shirt sleeves, breathing hard, and face flushed. He packed three valises and put them in the back premises of the next house during the evening.

"Later in the evening, towards night, he went to a hotel, ate supper, went to a barber shop and was shaved; returned to the hotel and played checkers until towards train time. Did not conceal the fact that he was on the eve of departure. To one he said he was going to Dallas; to another, San Antonio; to another, Georgetown; to another, Fort Worth. At the time named he went to the place where he had deposited his valises, obtained them, and made his way to the depot; remained in and around the depot until train time; train came in at about 11:40 p. m. Did not buy a ticket to Chicago, boarded the train, rode on it in seat with a party whom he knew and who knew him, conversed on different subjects.

"He was apprehended in Chicago about thirty days thereafter, and extradited for trial on charge of having murdered his wife and children. At the time of the murder he was out of business, without any ready means; judgment of forcible detainer had been rendered against him for the possession of the house in which he lived; process to oust him was in the hands of an officer, and the 24th of July was the last day he had permission of the owner to remain on the premises. [I will here state that he was under the impression that his bondsmen were going to give him up to the law to stand trial for forgery or embezzlement, for which he stood indicted, and his prospects for a long term of imprisonment were very strong. It is important to bear this in mind.] At no time anterior to the said July 24, nor on that day, nor on the day subsequent thereto, did he, to many friends and acquaintances, and those with whom he transacted business, present a demeanor, appearance, habits, or conversation different to what was usual with him."

The affection of the accused towards his family was a noted and remarked fact by those who knew them. He was indicted in one count charging murder of wife, the killing alleged to have been done with some cutting instrument; 2nd, by strangulation; and 3rd, by drowning. The plea of "not guilty" was entered. The defense was insanity; but no suggestion was made that the defendant was insane at the time of the trial.

A hypothetical question, based on the foregoing facts, was submitted to Dr. T. D. Wooten, his two sons, Drs. Joseph S. and G. H. Wooten, Dr. M. M. Smith, Dr. R. S. Graves, city physician, Dr. J. A. Davis, late assistant physician at the lunatic asylum, and Dr. B. M. Worsham, superintendent of the State lunatic asylum at Austin, witnesses for the State. They gave it as their opinion that on the night of July 24, 1896, Burt was sane. These gentlemen, or some of them, at the request of the State's attorney's, examined the defendant in jail, taking measurements of his head, testing the reflexes, etc., with the purpose of ascertaining his mental condition at the time of the trial, a question not at issue; and they gave it as their opinion that he was sane.

The hypothetical question embraced none of the facts elicited from witnesses for the defense, presently to be enumerated. When a hypothetical question, embracing exclusively the facts elicited from witnesses for the defense, was put to these same witnesses, they gave it as their opinion that on the night of July 24 Burt was insane. These facts were:

1. There was insanity in the family; it was hereditary; had appeared in grandfather and other members.

2. His mother, while pregnant with him, was wild, violently insane, and had to be restrained.

3. He was congenital moral pervert.

4. In childhood he was cruel, stole, lied.

5. As he grew to manhood he became alienated from his brothers, his only near relatives, and without cause.

6. Subsequently he became silent, morose; stole money, embezzled money, and committed forgery when there was no need of doing so; forging checks for trifling sums, $2 and $4.

7. He was devoted to his wife and children; had often been seen helping his wife in her household duties, even cooking; and he spent his evenings at home in preference to elsewhere, apparently preferring the society of his family to all other.

8. For the killing there was no ascertainable reason or cause.

Dr. R. M. Swearingen, State Health Office and Surgeon-General of Texas, Dr. J. W. McLaughlin, Dr. R. P. Talley, an uncle of the accused, physicians of large experience in general practice, and Dr. R. K. Smoot, a Presbyterian minister of Austin, were witnesses for defense. All of them had known the accused more or less intimately since his childhood. These witnesses gave it as their opinion that on the night of July 24 Burt was insane, Dr. McLaughlin qualifying his opinion by saying that he was "morally insane," a congenital "moral pervert." Dr. D. R. Wallace, of Waco, Texas, also summoned by defense, a physician of many years' experience in treating the insane, having long been superintendent of the State asylums at Austin and Terrell, and perhaps of all those summoned best qualified to give an opinion on the subject, gave it as his opinion that at the time of the killing defendant was of unsound mind. When asked if he was insane, he answered, "No, not insane, but of unsound mind."

That is a distinction without difference. All authorities agree that "insane" and "of unsound mind" are synonymous; that a person of unsound mind is insane. Professor Fisher says: "There is no distinction between 'insanity' and 'unsound mind.' "*

So that Dr. Wallace, though unintentionally, gave it as his opinion that Burt was insane the night of the killing. Nevertheless, his opinion, as worded by himself, had the moral effect of an opinion adverse to the accused, and was so accepted by the court.

It was also in evidence that defendant had been a bright boy. He had been brought up under good moral influences, his parents being eminently respectable Christian people, and he had had a happy home; yet at an early age he showed marked depravity; would lie and steal, and was cruel to dumb creatures; nailed a living rabbit to the ground, for instance. He was an affectionate son and brother. At the time of his father's death (his father had been a popular physician in Austin) in July, 1886, when this boy was sixteen years and nine months old, a marked change came over his nature and conduct. From a genial, happy member of a peaceful household, he suddenly became morose, taciturn, suspicious; held off from intercourse with the family; became alienated from his brothers, who are exemplary citizens of Austin, and who did all in their power to assist him in his misfortunes and pecuniary

---

*Witthaus and Becker's "System of Medical Jurisprudence."

troubles. They took him into their employment when he failed at all else, but he stole goods and money which he could have had for the asking; paid him out of several scrapes, and were on his bond at the time of this act, he being, as stated, under indictment for forgery or embezzlement. He regarded his brothers as his enemies, and had the belief that they had designs on his life. They had to send him away from their place of business.

A point here furnishes a link in the chain of presumptive evidence of insanity, which was not mentioned at the trial or brought to the attention of the experts. At the time of his father's death, when the first marked change in the boy's character was observed, he was in his seventeenth year, at the age of puberty, when any tendency to insanity is apt to be developed. So well is this established that the "insanity of puberty" is enumerated as one of the marked forms of the disease. In this case, with a strong hereditary predisposition, the marked change of habits and manners, taken in connection with the early evidences of a blunted sense, would appear to be a valuable diagnostic sign, which furnishes a link in the chain of the progressive development of the disease. A characteristic of this form of insanity is that the subject takes strong dislikes, especially to his nearest relatives.

The verdict of the jury was murder in the first degree, and the penalty death. An appeal was taken, and on June 9, 1897, the appellate court affirmed the verdict, and on May 27, 1898, the accused was hanged.

From the standpoint of the medical jurist, the jurisprudence of insanity is defective in at least three particulars:

1. The defendant in a case of the kind under consideration has not for the benefit of a diagnosis by the light of modern science, because recent discoveries and conclusions of medical science are not comprehended in the existing system. The laws have not been made to conform thereto, nor do the courts permit text-books, the standard authorities, to be quoted in support of alleged insanity.

2. The law leaves to the determination of a jury, often of unlightened men, metaphysical questions that baffle the ablest scientific minds, towit: the existence or non-existence of insanity, the degree of impairment of free will, and the extent of responsibility of a person adjudged insane by medical experts.

3. The courts do not exercise proper discrimination in allowing medical men to pose as experts.

1. We will show what the popular and generally accepted conceptions of insanity are, and the old pathology on which the system of jurisprudence is based, and compare it with modern conclusions as established by the latest authorities, on which, as I contend, a revised system should be formulated.

Professor Charles F. Folsom says:*

"The popular idea of insanity is of wild, incoherent, or crazy conduct. If maniacal, the timid, frightened young girl, who would not hurt a fly, and the tottering, harmless old man, if confused and partly demented, are hurried off to an asylum, * * * while the victim of overwhelming delusions, because he seems clear, logical, and collected, is vigorously defended against the physician's imputation of insanity, until he commits an offense against the laws, when he is fortunate if he is not treated as a criminal. It is often impossible for judges, juries, counsel, and even medical experts to wholly divest themselves of the popular notions of insanity in cases appealing strongly to the passions or prejudices of the day. Cases involving the question of responsibility for crime are decided against science and the evidence, because of certain preconceived notions of insanity which no amount of skilled opinion can controvert. Jurors and, less often, judges make up their minds what a sane man would do under given conditions, and of what an insane man is capable, judging from the facts within their own experience; and in forming their decision it is the act itself, and not the man, diseased or otherwise, in connection with the act, that chiefly governs them. * * * Strange, apparently purposeless, illogical, inconsistent action is frequently attributed to the author being insane on that subject, whereas he may be simply acting from a strong impulse or emotion, and may be by no means insane. On the other hand, because a man knows right from wrong in the abstract, and can ordinarily behave well, the very characteristic workings of his insane mind are often seized upon as unquestionable proof of sanity, even when admitting of no other explanation to the skilled physician than that of insanity. * * * With precisely the same degree of insanity, and the same power to control their actions, two murderers may be sentenced, the one to death for an act where the motive and method were those of the criminal, and the other to an insane asylum for killing a person under circumstances which are not explainable by sane reason."

Buchnill says:†

"It is a trite but most important observation that in the question of what constitutes insanity, the members of the two great and learned professions, law and medicine, entertain essentially

---

*Pepper's "System of Medicine."

†"Insanity in its Legal Relation."

different and seemingly irreconcilable views, and that on the question of the irresponsibility of criminals who are supposed to be insane, there is a wide chasm of difference between them. To a certain extent this is true, and perhaps inevitable; and the reason for it is not hard to find: that the two professions have to regard insanity and to deal with the insane with different aims and purposes—the physician to prevent and cure, the main question with him being to prevent its interference with the duration and enjoyment of life. To the lawyer * * * the sole question is its existence, its degree, and its influence on the conduct; it is, with him not a medical question, but a moral one. * * * The degree of loss of free will is a question for the jury; the fact that the will is impaired is for the expert to establish. * * * A person may be insane medically, yet not in the eye of the law. It is for the jury or experts to determine the fact of insanity; the courts to determine its effects on civil rights."

Like the shield which to one observer was golden, and to the other argent, insanity presents itself in different aspects according as it is regarded from one standpoint or another.

"Our conception of mental disease," says Professor Fisher,* "depends entirely whether we look at it from a medical or a legal standpoint."

Ray says (*ibid*):

"Insanity in medicine has to do with a prolonged departure of the individual from his natural mental state, arising from bodily disease. Insanity in law covers nothing more than the relation of the person and the particular act which is the subject of judicial investigation. The legal problem is, whether there was mental capacity and moral freedom to do, or abstain from doing, that particular act. The general meaning of insanity in law is, a permanently disordered state of the mind, produced by disease, and beyond the control of the individual."

Professor B. Sachs says:†

"Very few legal minds have been able to get beyond this antiquated view of the relation of insanity to crime. In Germany and France the more intelligent judges have been guided by the opinions of the medical experts, but even there they are not bound by such opinions; and it has happened time and again that the judge, having asked for and received the opinion of the expert, has promptly set it aside and decided the question to the contrary.

---

*Witthaus and Becker's "System of Medical Jurisprudence."

†Insanity and Crime; Hamilton's "System of Legal Medicine."

* * * This right and wrong test has been the stumbling-block in the advance of legal psychiatry; and, as a matter of fact, if the test were applied to the insane (in an asylum), nine of every ten would have to be declared sane, for the most of them are perfectly aware of the nature of the acts they commit; the majority of them know that they are right or wrong according to the ordinary standards; but they are impelled, either by sudden influences or by sudden forcible delusion, to the commission of acts which they know to be wrong, and which they, if sane, would never have committed."

Witthaus and Becker says:*

The knowledge of right and wrong is not a fair criterion, as many insane men possess that knowledge well enough in the abstract. * * * A man may know right from wrong, and yet not have the will power to abstain from doing what he knows to be wrong."

Dr. R. M. Bucke, superintendent of the largest insane asylum in Canada, with a view to determine this question, canvassed the 1034 inmates, and found that 763 "were perfectly capable of realizing and appreciating such an act as homicide in its moral and legal relations." In other words, "nearly three-fourths of the inmates were responsible and fit subjects for capital punishment as the law now exists." (See report for 1896.)

Dr. John B. Hamilton, superintendent of State Lunatic Asylum of Illinois, in the *Journal of the American Medical Association*, says:

"The legal standard of responsibility (knowing right from wrong) given in the famous answer of the judges to the House of Lords in connection with the celebrated McNaughten case, * * * which has been adopted in this country, has always, from the first, had the disapproval of competent alienists, those who of all men are best qualified to estimate the responsibility of the mentally defective. They have used every argument against it; have proved that it is a false criterion in almost every possible way, have shown clinically and pathologically its incorrectness, but have not as yet been able to thoroughly eradicate the belief in its validity from the legal mind."

He stigmatizes it as "irrational barbarism." The celebrated alienist Dr. Morel was seized with an irresistible impulse to throw a working man into the river, and fled from the spot to prevent doing so. Numerous cases are recorded illustrating this lack of power to resist an impulse, knowing it to be wrong; no fact is

---

*"System of Medical Jurisprudence."

better established in the whole science of criminology. In works on medical jurisprudence a case is related of a woman who had an impulse to kill her children and asked to be locked up. Such cases are innumerable.

To many persons the sight of a sharp instrument prompts the desire or impulse to kill some one; and there are persons who dare not carry such weapons for fear they may do themselves or friends harm. Burt, when arrested, asked the sheriff to take his knife, "for fear he might hurt some one." There is, indeed, a form of insanity called "reasoning insanity," in which the person understands what he is doing and the true relation of the act in its social and legal aspects. "He, however, prefers the consequences to the restless, unhappy state of mind that exists until it is done." (Dr. Hamilton, *ubi supra.*)

The greatest advances in the study of mental diseases have been made within the last quarter of a century. Within that period medical science has realized that insanity is a manifestation of disease of the brain (though disease of the brain is not necessarily insanity) ; that the brain, the organ of the mind, is the seat of the disease; and that there can be no such thing as partial insanity. A man is insane or he is not insane, as he may be sick or well; but it is a matter of degree. Thus, for the first time in the history of medicine has there been a scientific basis for insanity; and the study suggested by this view has enabled alienists to formulate a rational classification of the disease. In that time, too, a new science has been born, the science of criminology, or criminal anthropology; and those cases known to alienists as "borderland cases," so-called moral insanity, a condition between insanity and depravity, and barely distinguishable, if at all, are now recognized as forms of congenital madness. Gorofalo was the first to differentiate them, and to him belongs the credit of defining their characteristics. Lombroso, Gorofalo, Ferri, and others of the new school describe these as "congenital delinquents," "degenerates," "natural insane criminals"; and with painstaking care Ferri* has pointed out the distinguishing features of each class. Lombroso and his followers have even formulated a set of physical defects or marks—"stigmata"—as distinctive and diagnostic.

This new school classifies criminals into: (1) The madman born (the born murderer is a born madman) ; (2) The homicide by occasion; (3) The homicide by passion; (4) The habitual homicide. None of these concern us except the first, the natural

---

*Enrique Ferri on "Homicide," vol. 2.

criminal, who is always mad. He is born to kill; and, given the opportunity and the impulse, he can no more help killing than a stone can help falling when thrown into the air; he kills in obedience to an impulse for which he is not responsible, and which he can not control.

In the congenital criminal insane (mark the distinction between "criminal insane" and "insane criminal," the one being born insane with homicidal impulses; the other being a criminal who has become insane.—Flint) the most marked psychological characteristics, as pointed out by Ferri (who uses for this class the synonym, "congenital delinquent," or "born delinquent," that is, the victim of an hereditary predisposition to insanity, with homicidal impulses), are: moral and physical insensibility; insensibility toward the victim, toward the sufferings of others; a cold ferocity in the execution of the crime; an apathetic impassiblity after committing the crime, and even at the sight of the victim; quiet sleep after the deed; impassibility to their punishment, and indifference to death, often resulting in suicide. This ferocity, this indifference, says Ferri, this insensibility of the born homicide, serves as a psychological explanation of other characteristics conjoined to these. The indifference is chronic, manifesting itself in preoccupation with most trivial things, which can not be attributed to corruption during confinement. (Note Burt's trifling conduct in prison: his putting on a mask and charging a fee to show his face, etc.). They feel no repugnance to the idea or to the act of homicide; they have no moral sense; they have no remorse concerning their offense. "To this absence of remorse must be added stubborn denial, indifference as to escaping punishment, and the easy adaptation to prison life."

"Altruistic sentiments," says the author, "such as love, family affection, etc., are not lacking in the congenital mad homicide. They are not even incapable of noble actions, but their immoral temperament renders them unstable, contradictory, and thus that same altruistic sentiment may find expression in their very crime."

The fundamental psychological characteristic he defines thus: "An abnormal impulsiveness of action, for lack of, or owing to weak power of resistance to criminal desires; a normal man subject to such impulses can resist them." He cites also the case of Dr. Morel and other cases. The congenital mad homicide can not thus defend himself. These facts are due, he says, to congenital weakness or arrest of development; such defectives are not apt, not educated, to resist. Of the psycho-pathological symptoms of the congenital mad homicide, Ferri says: "The deliberations of this unhappy person are due to either a slow invasion of the homicidal idea" (which he calls "homicidal obsession") or "momentary impulse." Hence, two distinct generic types of psycho-pathological characteristics.

In the first type the desire to commit crime "springs from a slow and reflective process, which increases from the weak or static state (obsession) until it becomes an irresistible impulse and takes violent and dynamic form finding vent in the criminal act. Sometimes he has a perfect cognizance of his own madness, of the act he intends to commit, and of the punishment due to it; nevertheless this will not, can not, deter him unless external or fortuitous causes intervene." The madman affected by homicidal obsession is incapable of restraining himself. This author cites the case of a man who, unable to dominate the violent force impelling him to murder his wife and children, consigned himself to the police and had himself locked up.

In the second type the determination to homicide "proceeds from a spontaneous impulse" (as was Dr. Swearingen's opinion in the Burt case), the "transitory mania" of the old school of psychiatry; "impulsive insanity" (homicidal) of the newer; "impulsive vertigo," without a real motive.

Perhaps the most significant characteristic distinguishing the born murderer (congenital mad delinquent) from the murderer by habit or occasion, as pointed out by Ferri, whose work may be taken as the exponent of the latest teachings on insanity and crime, is that, whereas the latter has always some selfish purpose or benefit in view, antisocial in its nature, murder being a means to that end, with the congenital criminal insane (of which class I regard Burt as a striking illustration) the murder is itself the end; killing to kill, impulse without motive, or as "a means to an end more often social or juridic"; that is, "as a defense of the victims from misery or want, or a worse fate."

Still another characteristic of the born insane homicide which Ferri names, is that he is possessed with the idea (obsession) to sacrifice the victim for his own good, or for the good of both self and victim. I have not the slightest doubt that Burt intended to complete the tragedy by suicide, but that either he was interrupted by some circumstance, or the obsession passed off before he effected his purpose. Ferri also says of the congenital mad murderer, that his previous conduct is often regular, when suddenly, some time before the murder, a change of life and character takes place. Striking characteristics are: his attitude during trial; his protests that he is not mad; the dissimulation of his insanity, or even his simulating another form or madness than that from which he suffers; non-resistance to arrest; no attempt, or a silly one, to escape.

"The absence of any real motive," says Professor Fisher,* "the history of hereditary taint, a neurotic disposition, seem to establish proof of mental weakness, at least approximating the confines of insanity."

Mr. Louis E. Binsse says:†

"Evidence of the want of motive on the part of the accused for the perpetration of the deed is considered to be a strong corroboration of the fact of irresponsibility."

Chief Justice Hornblower (State *vs.* Spencer, N. Y.) says:

"I do not say that the absence of apparent motive invariably exists in cases of homicide committed by insane persons, but I say it generally is the case."

"Motiveless homicidal ideas occur to husbands and wives and parents with reference to those dearest to them under conditions of prolonged mental strain." (Witthaus and Becker.)

Statistics show that killing of near relatives by the congenital mad homicide occurs eight times oftener than that of any other." (*Ibid.*) "A crime performed without accomplices, with no plan or a silly one for escape, and no sane motive, is usually itself evidence of insanity." (*Ibid.*)

The last rational act Burt is known to have done on the night of the tragedy was to take his baby from the arms of the nurse, while the mother took the elder child, fill its bottle with milk, feed it, undress it, and get it to sleep. Within an hour or so he brained it and the others with a hatchet. Was that the act of a sane man? He packed and shipped the bloody garments and the hatchet to Houston; he went to Chicago and mingled with the people in the most public place, the Board of Exchange, meeting there acquaintances who recognized him, yet returned there again and again, knowing that a reward was offered for his arrest. Was that an effort to escape? The State asserted that there was a motive, but the best they could offer was "the proceeds of the sale of the furniture, $65." That is too absurd for serious consideration.

2. The most unjust and pernicious feature in our system of jurisprudence in the adjudication of cases like Burt's is that which leaves to a jury the determining of the question of the existence of insanity in the accused, the degree of impairment of will power,

---

*Witthaus and Becker's "System of Medical Jurisprudence."

†"Theory of Criminal Responsibility."

and his responsibility to the law. Where the opinions of the medical experts as to the existence of insanity clash, as they almost always do, it is left to the jury to decide. As the average juryman is not usually of a high order of intelligence,—indeed, in some cases the juryman is selected because of his want of knowledge, ignorance being a qualification to serve,—the absurdity of the law is apparent.

A fact is something that can be demonstrated. The best informed alienists can not state as a fact that insanity exists in a given individual; its existence is a matter of opinion, of judgment, the result of a process of *a posteriori* reasoning, a conclusion arrived at from weighing all the evidence, from comparing the relation of facts one to another, and their bearings. The average juryman has not the faculty to thus reason, because, no matter how high his native intelligence, his mind has not been trained by study. The differences of opinion between medical expert witnesses mark the differences in their grade of intelligence and learning, as well as in their power to reason from effect to cause. The medical man with an analytical mind, vast learning and experience, is not liable to reach the same conclusion on a metaphysical subject, even with the same facts before him, as one of a different order of mind, or of less experience or reasoning power. Hence the differences between expert medical witnesses, so often ridiculed, are not so illogical when looked at in the light of cultivated intelligence. It is peculiarly the mission of medical science to discover the cause of disease. Insanity is a disease, and, as such, is as much the exclusive province of the medical man as is smallpox. It requires more ability to recognize occult mental disease than any other pathological condition, and yet our system of jurisprudence relegates these intricate questions to the verdict of jurymen profoundly ignorant of everything pertaining to the case. It is as illogical as to call in a layman to decide a point of diagnosis when two medical consultants have differed. "If left in doubt," says Dr. Sachs, "the jury generally decides on its own impressions; and, if in time of general excitement, usually decides against the accused whose defense is insanity." They have no other method of deciding.

When, then, shall the plea of insanity be considered valid in extenuation of crime? "The only proper answer to this question," says Dr. Sachs,* "in the light of the present condition of psychiatry, is that no person shall be considered guilty of crime if, at

*Hamilton's "System of Legal Medicine."

the time the crime was committed, he was suffering from any form of mental disease." New York has practically made her statute accord to this. The statute says: "No act done by a person in a state of insanity can be punished as an offense." Again Dr. Sachs says (*loc. cit.*): "All nations agree in absolving from responsibility a person of unsound mind." In pursuance of the amended law, Judge Gildersleve, in the Appellate Court of New York, charged the jury in the case of People *vs.* Mrs. Lubinaker: "If a reasonable doubt exists as to whether the prisoner is sane or not, she is entitled to the benefit of the doubt, and to acquittal." And this is the law in most States. In Burt's case, Judge Brooks gave the jury the law to that effect.

And here I will ask, can any rational man, acquainted with the facts in Burt's case, say there was not a reasonable doubt of his insanity on the night of July 24, if, indeed, his insanity were not established by a preponderance of medical opinion? In Hamilton's "System of Legal Medicine," Dr. Sachs says: "The medical expert should be called upon to state whether the accused is or was sane or insane; and if insane he should not be held responsible for his acts.

There is a unanimity of sentiment on this head. Hence, the important point to be established is, the existence or nonexistence of insanity in the accused. As any departure from a physiological state, however slight, is pathological, so, given a standard of mental sanity, any deviation from that standard, however little, is an abnormal state, that is, insanity. Hence, there are innumerable shades of mental unsoundness, merging the one into the other, ranging from slight alienation to violent, raving mania. No doubt there are hundreds of insane people amongst us, walking the streets and attending to the affairs of life, who are liable to an explosion of insanity at any moment, but who, until such explosion is brought about by developing causes, are never suspected of any unsoundness. Oliver Wendell Holmes, in "The Autocrat of the Breakfast Table," said that the worst cases of insanity are those outside of the insane asylum. Haslam, in his day one of the first medical experts in England, declared in open court that he had never, in the whole course of his life, seen a sane person. And there is a growing tendency on the part of the medical profession to regard all crime as manifestations of mental alienation.

It is absolutely essential, therefore, that a midway position should be determined upon, a line drawn, where responsibility ceases. But to make any such line hard and fast is an absolute impossibility; it must needs be, in the very nature of things, more of less flexible; no rule of the kind can apply to all cases, or to all forms of insanity. Common sense, reason, and justice demand that the determination of such a question should be left to the ablest and most experienced students of mental disease.

Observe the inconsistency of the law. It is universally held that sanity is an essential requisite to crime. It is a maxim of law that an insane person can not commit a crime. Said Judge Hurt, of the Texas Court of Criminal Appeals, by which the Burt case was finally decided, in the case of Levi King *vs.* State:

"What sane mind can comprehend the possibility of a crime being committed by an insane person? If the prisoner is insane, there is no crime. If there be crime, there is no insanity. Insanity can not excuse crime, for the fact that, if insane, there is no crime to be excused."

That is the law. It is unqualified. Nothing is said of any degree or kind of insanity; it is sufficient that the party is insane. It is the province of the medical man to prove the existence of insanity, and yet in every State except New York—and that in consequence of recent reform—it is the rule for the court to charge the jury to determine the degree of insanity, the degree of impairment of will power, and the responsibility of even a person proven by unanimous medical opinion to be insane. And to do this they are instructed to apply the antiquated and misleading test of knowing right from wrong. In effect the law says: "True, Mr. Expert, you say the accused is insane; admitted; but hold on; let us see *how* insane he is. Is he so insane that he does not know right from wrong? It is for you, Mr. Juryman, to determine that point." In the name of all that is consistent, how can a jury of often ignorant laymen determine such a question?

In Burt's case it was objected by the State that to leave the determining of the existence of insanity, the degree of will-impairment, and the responsibility of the accused to medical experts would have been tantamount to an acquittal: "insane," *ergo* "irresponsible," *ergo* "not guilty." Be it so. It would be a wiser and more just course than that now pursued.

A solution to this difficult problem would be to have a Medical Court in every State, paid by the State, to whom should be left the adjudication of all points of medicine in its relation to law; just as we have courts of law to settle all legal points. Trial by jury is a relic of barbarous ages, and has degenerated in a large number of cases into a travesty of justice. If accused of crime I would rather trust my fate to the toss-up of a penny than to stand trial by a jury to whom is given the determining of questions so far beyond their powers of comprehension.

3. The courts do not exercise proper discrimination in permitting medical men to testify as experts.

"Much of the disrepute into which hired testimony has fallen," says Dr. Hamilton,* "is undoubtedly due to a kind of partnership which many men find it difficult to avoid; for the engagement of their services implies a bid for help in advancing a side by the building up of theories for the support of a more or less tenable position. * * * If an expert be careless of his reputation, or weak or corrupt, he will lend himself to the side of the case upon which he has been retained, and in reality he becomes a pleader."

Again, Dr. Hamilton says:

"That there is need for reform is undeniable, and that the courts do not exercise sufficient care in fixing the status of medical witnesses is equally true. The strictures of medical writers, courts, and others are just, so far as the existence of demoralization goes. As the law is administered many persons can be found who are ready to arrogate knowledge and position they do not deserve. The dignified alienist of experience and reputation is confronted by the imposter, whose glib manner and bizarre 'popular-science' learning sometimes impress the susceptible juryman as does the proprietary-medicine advertisement, and whose experience of medicine and its exponents is confined to the quack or cure-all. The law is largely responsible for this."

Says Dr. Sachs on this subject (*loc. cit.*):

"Psychiatry is a very special branch of medicine. It does not constitute a part of the regular medical training in this country; yet, in some of the most important trials of recent years, and medical man has been accepted as an expert, and his opinion has been held to be fully as valuable as that of a man who has devoted years of study and practice to this special branch."

There is something strangely illogical, arbitrary, and absurd in a rule which excludes the teachings of the ablest alienists and the latest conclusions of investigators in the field of mental disease,—books in which are vividly drawn the clinical features of each type of insanity,—and disallows the citation of authorities as to the distinguishing characteristics of the disease; yet allows totally inexperienced medical men, who have never treated or observed a case of insanity,—"sophomore experts," Major Walton, Sr., counsel for defense, calls them,—"to read up on authorities there

---

*"System of Legal Medicine."

is no telling how old, and then rattle off their interpretation of the text as their 'opinion.' "

It is difficult for a medical witness not to share in the sympathy for or against a prisoner, and to be influenced by popular prejudice. In a case like Burt's, where the feeling against the unfortunate man was so strong, it required a brave man to run counter to popular clamor. Such a man makes himself unpopular, and unpopular means loss of patronage. One feature of rank injustice done to the prisoner was permitting men to pose as experts who had never even seen a case of insanity, and were by no means expert in ordinary medicine, much less in mental pathology, and giving to their opinion equal weight with that of the alienist by profession and experience. Some of these witnesses, moreover, seemed influenced by the popular prejudice manifested by the audience, for part of their testimony was given in such a way as to appear to be intended to meet popular approval, and to suggest "playing to the gallery."

A review of all the facts connected with this sad affair forces the conviction is my mind that the defendant Burt was at the time of the murder, and had been for years, insane. His case corresponds in every detail to that form of hereditary insanity which is developed gradually until it overpowers reason and leads to crime. That the verdict in his case was not just, and not in accord with the evidence, I firmly believe. O, Justice, how many cruel wrongs are perpetrated in thy name!

Had the symptoms and all the acts of the defendant been detailed to the medical witnesses, the better-informed of them could hardly have failed to diagnose a well-marked type of the criminal insane degenerate of Lombroso, a born criminal of the class demonstrated by him to be always morally insane. Almost every feature in the case tallies with the characteristics of the natural criminal insane with homicidal impulses, as described by most recent writers. Its counterpart could have been found in many recent works, had the court permitted them to be cited. Had counsel been allowed to read to the court and jury the clinical picture of the born insane homicide, so forcibly drawn by Ferri, and quoted above, no man of ordinary intelligence, knowing the facts in the case, would have failed to recognize Burt in the picture, as a strong illustration of that type of the insane.

A parallel case, to which I have referred, is that of Mrs. Lubinaker. Poor, in very bad health, a widow, eating, and feeding her three children only as she was able to earn money to buy food, pregnant and shortly to be confined, she thought she was going to die, and the thought of her children starving prompted her to kill them. No remorse, no concern for the consequences; she realized that she had committed a crime in law, but her only idea was that they would be better off in another world. She intended to commit suicide, and dividing the poison, "rough on rats," in four parts, one for each child and one for herself, she gave it to the children. Two of them died, but the sufferings of the other one diverted her mind from killing herself. She went for a doctor, not to save the child,—she did not think of that,—but to relieve its sufferings; and in that way she was prevented from completing the tragedy. At the trial she was convicted, being pronounced "sane" by the jurors. But in the higher court, expert testimony—Dr. Allen McLane Hamilton and other equally celebrated alienists—pronounced her insane, of the type here being considered, and she was acquitted.

Ferri describes a type of the insane, as above cited, who have killed their children to save them from want, in whom one strong characteristic is lack of emotion, indifference even at the sight of the corpse of the victim. In this connection is recalled the stoic indifference of Burt during the trial, when the bloody hatchet and the garments of his murdered innocents, stained with their blood, shed by himself, were exhibited to the jury. He sat as one dazed, as senseles as a stone. If he were "acting a part," as was said by some of the "experts," it was a masterpiece of acting. His stoicism would have done credit to a savage. By most of the experts he was said to be "simulating," *itself a distinguishing feature of a now well-recognized form of insanity.* The alleged experts were unable to interpret the signs, and attributed his insensibility to a display of "nerve"; and it added to the prejudice of the populace.

So flagrant was the deed, so horrible; so seemingly rational was the conduct of the unfortunate man, both before and after the deed; so methodical seemed all his acts, that few would believe but that there had been a deliberately planned murder, notwithstanding no one could even conjecture a reason or motive for it. Prejudice ran high, the people were strongly arrayed against him, and the plea of insanity was fairly laughed at. The audience were in sympathy with the State witnesses. When damaging testimony was elicited a visible throb of exultation ran through the crowd. Their desire for a conviction and death sentence was so manifest that they were threatened by the court with expulsion.

One medical witness was asked: "Do you understand the workings of the human mind?" He replied, "I do." He is doubtless the only human being thus gifted, and he should have been asked to analyze the thoughts that passed through that miserable creature's mind, the emotions that struggled in his breast, that night, as he gazed upon his sleeping innocents and realized that on the morrow grim want would thrust them from beneath the roof that sheltered them, out into the streets—beggars; he, their father, a man of some education and refinement, who had been raised in comfort, if not luxury, ostracized, denied work, without money to buy bread, without friends, without resources of any kind, momentarily apprehensive of arrest and imprisonment. Ah! it would not require the genius of our gifted medical mind-reader to divine that the thought dominant in his mind was, "What will be the fate of my loved ones, my two little daughters, when I am sent to prison, perhaps for a long term?" "Cast into this breathing world scarce half made up," mentally deficient and morally weak, heir to a propensity to evil, hedged in by a combination of most distressing circumstances, enough to have dethroned a reason more firmly seated, is it strange that the impulse seized him to end, then and there, the unequal struggle? to kill his loved ones to save them from a worse fate—kill them *because* he loved them?

Our penal system is based upon the ancient law, "an eye for an eye, and a tooth for a tooth." Vengenace appears to be the chief end; retaliation rather than justice. The basis of our system is a police regulation formulated to meet the exigencies of a barbarous nomadic race two thousand years ago, and not adapted to the requirements of a latter-day civilization. The law should have for it object something higher than revenge. "Our system of jurisprudence," says Dr. Wines, "should not only be humane, it should be intelligent." The protection of society, the deterring of criminals, and the lessening of crime are the ostensible objects of capital punishment. It is a demonstrated failure. The ends can be secured by means less revolting.

It is argued that, from an economic standpoint as well as for the protection of society and future generations from the evils of the hereditary transmission of criminal propensities, it would be best to exterminate this class of offenders; they are worthless to the world and to themselves; their lives are blighted. Why not hang them? To do so would be most expedient—*if we were savages.* But humanity revolts at the idea of executing an irresponsible creature; it is inhuman. The escutcheon of this free and enlightened government is already stained indelibly with the blood of too many irrational creatures, imbecile paranoiacs. In lieu of death, it is suggested that emasculation and perpetual confinement at whatever labor they may be capable of performing would be much more rational and humane, and would effectually cut off hereditary evils, thus affording the protection aimed at by the more brutal method in vogue.

In estimating responsibility it should be borne in mind that the warp in the physical, mental, and moral make-up of a defective antedates even his intra-uterine life. We have heard the saying that, "To reform a drunkard, you must begin with his grandfather." The blight is in the germ that fertilizes the ovum, which becomes, first embryo, then child. Hence, we have born into the world everything human, from the acephalous idiot to the godlike Robert E. Lee or Gladstone. Thus are the sins of the father visited "upon the children even unto the third and fourth generation," and all successive generations. Such defectives are no more responsible, morally at least, for their character and actions than they are for being here at all. The true philosophy of the situation is that, as far as possible, such defectives should be *prevented.* A decent regard for race integrity, to say nothing of present protection, demands it; and if our marriage laws were properly amended and enforced, and the services of the surgeon were utilized as above suggested, there would, in a short while, be fewer Guiteaus, Prendergasts, and Burts to puzzle and confound our learned jurists.

I am well aware that any hope of instituting radical changes in a system so universal and so long established is utopian. But were everybody content with existing conditions, there would be no progress in any department of human activity,—in law, medicine, art, science, literature, finance, or commerce. No errors would be corrected or evils eradicated. Hence, when a human life so often depends upon rules of court based upon an antiquated conception of insanity, it is needful to insist that the voice of science shall be heard, and that the great truths revealed by laborious investigation and experimentation—truths vital to the dearest interests of mankind—shall be utilized in medical and criminal jurisprudence. Our system needs to be remodelled, made more comprehensive, and adapted to the changed condition of the knowledge of insanity and to the demands of an advanced civilization.

